# Compositional Analysis and Mechanical Recycling of Polymer Fractions Recovered via the Industrial Sorting of Post-Consumer Plastic Waste: A Case Study toward the Implementation of Artificial Intelligence Databases

**DOI:** 10.3390/polym16202898

**Published:** 2024-10-15

**Authors:** Federico Olivieri, Antonino Caputo, Daniele Leonetti, Rachele Castaldo, Roberto Avolio, Mariacristina Cocca, Maria Emanuela Errico, Luigi Iannotta, Maurizio Avella, Cosimo Carfagna, Gennaro Gentile

**Affiliations:** 1 Institute of Polymers, Composites and Biomaterials, National Council of Research of Italy, Via Campi Flegrei, 34, 80078 Pozzuoli, Italy; federico.olivieri@ipcb.cnr.it (F.O.); antonino.caputo@unina.it (A.C.); rachele.castaldo@ipcb.cnr.it (R.C.); mariacristina.cocca@ipcb.cnr.it (M.C.); errico@ipcb.cnr.it (M.E.E.); avella@ipcb.cnr.it (M.A.); minocarfagna@gmail.com (C.C.); gennaro.gentile@ipcb.cnr.it (G.G.); 2 Lavorgna Igiene Urbana srl, Via Tratturo Regio, 82030 San Lorenzello, Italy; laboratorio@lavorgnasrl.it (D.L.); igieneurbana@lavorgnasrl.it (L.I.); 3 CRdC Nuove Tecnologie Scarl, Via Nuova Agnano 11, 80125 Napoli, Italy

**Keywords:** polymers, post-consumer plastic waste, plastic sorting, fractions, mechanical recycling, artificial intelligence

## Abstract

Nowadays, society is oriented toward reducing the production of plastics, which have a significant impact on the environment. In this context, the recycling of existing plastic objects is currently a fundamental step in the mitigation of pollution. Very recently, the outstanding development of artificial intelligence (AI) has concerned and continues to involve a large part of the industrial and informatics sectors. The opportunity to implement big data in the frame of recycling processes is oriented toward the improvement and the optimization of the reproduction of plastic objects, possibly with enhanced properties and durability. Here, a deep cataloguing, characterization and recycling of plastic wastes provided by an industrial sorting plant was performed. The potential improvement of the mechanical properties of the recycled polymers was assessed by the addition of coupling agents. On these bases, a classification system based on the collected results of the recycled materials’ properties was developed, with the aim of laying the groundwork for the improvement of AI databases and helpfully supporting industrial recycling processes.

## 1. Introduction

Plastic products are highly predominant in several sectors because of their interesting performances and effective costs with respect to other materials [1]. The typical characteristics of plastic products include the good optical, thermal and barrier properties, good mechanical performances, light weight and easy processability [2].

Amongst the different plastic products, packaging is one of the most important applications [3]. The main function of plastic packaging is the protection of different products, aimed at preventing damage during their transport and storage and at increasing their useful life. This concept of protection is even more important in the case of the food packaging industry, with the main scope of food packaging being to delay the deterioration of food and to guarantee its high quality and safety by providing chemical, physical and biological protection [4].

Nevertheless, such packaging materials are becoming the major component of the plastic waste stream because of their short life cycle, thus seriously contributing to overburdening the management of urban solid waste disposal [5].

Indeed, urbanization, economic development, and the increasing population are continuously increasing the use of plastic products, with the consequent progressive growth of the generated plastic waste [6]. At present, the estimated worldwide yearly production of municipal solid waste has reached the incredible value of about 2 billion tons per year, and this figure is expected to increase by roughly 70% by 2050 [7]. Within this global figure, in 2020, the worldwide plastic waste production was estimated about 400 million tons, with about 8.8 million tons mismanaged and entering the oceans [8].

At the end of their useful life, plastic wastes are discarded and, coming into contact in different ways with various ecosystems, they can have a negative impact on the environment and human health through different mechanisms [9,10,11]. Indeed, plastic wastes can act as carriers of organic pollutants and heavy metals [12,13,14,15] and also of various pathogens [16,17]. The progressive degradation of plastic products under the action of physical, chemical and biological processes gives rise to smaller plastic debris, so that in the last few years microplastic pollution has become a global environmental problem of emerging concern [12,18,19,20], also because they can interact with biological systems and have relevant negative effects on human health [21,22,23,24,25].

To effectively deal with the increasing quantities of plastic waste, in addition to other objectives, such as the reduced production of plastic waste, the harmonization of national measures on packaging and the management of packaging waste, the Packaging and Packaging Waste EU Directive promotes the reuse, recycling and other forms of recovery of packaging waste, instead of its final disposal. In particular, with this Directive, the EU sets the following specific targets for plastic recycling: 50% recycling rate by the end of 2025 and 55% by 2030 [26].

In this frame, the most effective strategy for the management of post-consumer plastic waste is considered a holistic approach that include effective waste collection schemes, sustainable sorting processes and finally, mechanical recycling of recovered homogeneous plastic fractions [27,28,29].

Mechanical recycling is a complex process including the grinding of the waste items, their washing and, for thermoplastic polymers, their reprocessing by melt mixing, in particular by extrusion, to produce new secondary plastic compounds that in general retain their original chemical structure [30]. Nevertheless, for mechanical recycling, it should always be considered that most of the polymers constituting plastic waste streams are immiscible.

Thus, the simultaneous reprocessing of inhomogeneous plastic fractions containing different types of polymers leads to the production of recycled compounds with poor mechanical properties, mainly due to the potential phase separation phenomena of the different polymeric components. For this reason, the compositional heterogeneity caused by the complex composition of specific objects (fillers, additives, multilayered structures) and/or contamination by organic and inorganic substances during the life cycle represents a major technological challenge for recycling in terms of the quality and properties of recycled materials [31,32,33,34,35,36,37].

Thus, the effectiveness of sorting processes, that is, their ability to reduce the inhomogeneity of plastic streams to be reprocessed, and the implementation of methods to identify and quantify contaminants are highly relevant parameters to evaluate and improve the economic sustainability of post-consumer plastic recycling strategies [38].

To effectively sort plastic wastes, different approaches are currently used. Amongst them, the main strategies are based on source separation or post-separation. In source separation processes, citizens are required to separate plastics from other wastes before their collection, while in post-separation, wastes are separated after collection, mostly at treatment and recovery centers. In Italy, the source separation approach is used, where citizens separate plastic items from other classes of wastes. Then, the sorting of the multicomponent plastic waste stream is performed in industrial sorting plants to obtain homogeneous fractions constituted by the same types of polymers, each of them suitable for mechanical recycling. With the currently available technologies, the main recovered fractions are those constituted by poly(ethylene terephthalate) (PET), polyethylene (PE), polypropylene (PP) and polystyrene (PS) [39].

Moreover, one of the significant barriers preventing higher recycling rates is the variability of the properties of recycled plastic products, deriving from the variable properties of the polymers constituting different items in the original plastic waste streams [40,41].

In this frame, it can be inferred that the prediction of the most suitable process conditions to obtain the most performing product after recycling is not trivial. Nowadays, digitalization supplies the growing need for viewable data to streamline the processes’ performances. Some research investigated how big data interpretation and artificial intelligence (AI) can support industrial recycling to allow the realization of relatively high-quality recycled materials [42,43,44]. Indeed, AI can be used to automate processes such as sorting and waste management: AI algorithms have the potential to identify and sort different recyclable polymers with excellent efficiency and accuracy, and AI-assisted recycling facilities could streamline manual labor and operations generally. Then, AI algorithms, by analyzing data and patterns, and therefore, by predicting and planning the best methods for the recycling of heterogeneous materials, can optimize recycling processes, reducing waste. AI also has potential in the revolution of recycling infrastructure and logistics by analyzing data on specific collection routes and recycling facility capacities in terms of the optimization of the collection and transportation of recyclable materials. These algorithms can be based on many different machine learning methods, including neural networks [45], support vector machines (SVMs) [46] and k-nearest neighbor (kNN) [47]. Overall, AI can potentially enhance all of the recycling system, reducing its costs and minimizing its environmental impact [48]. However, as said, the boundary conditions may vary by analyzing a specific social fabric; so, the analysis and the interpretation of further cases of study have the potential to further increase the knowledge, helping to implement strategies and structures, improving their functionality in optimizing recycling processes.

In this work, we performed a case study investigation that included the compositional analysis of different batches of multi-material post-consumer plastic wastes collected in an industrial sorting plant in Italy, the characterization of homogenous plastic fractions after the industrial sorting process, and the evaluation of the processability and performances of recycled compounds obtained from different plastic waste batches. Moreover, we investigated the effectiveness of performance improvers in terms of the properties of the recycled compounds. Finally, in view of facilitating the implementation of AI databases to improve the efficiency of recycling processes of polymer wastes, we hypothesized a classification method for the recycled compounds that is able to easily classify them in terms of the processability and performance.

## 2. Materials and Methods

### 2.1. Analysis of Unsorted Plastic Waste

Three different batches of light multi-material plastic wastes were collected at the industrial sorting plant Lavorgna Igiene Urbana srl (San Lorenzello, BN, Italy) and characterized to evaluate the average composition of the unsorted materials. Before characterization, the collected wastes were washed with deionized water for 1 h at 90 °C and allowed to dry in environmental conditions (25 °C, 50% RH) for at least 2 days. Cross-contamination amongst the samples was prevented by careful storage of each batch. Each batch was stored in a different plastic container. The washing system was cleaned 3 times with deionized water at the end of each washing process, before passing to the washing of the next batch.

Each collected item was weighted, then a small fragment was manually removed and the material was characterized by Fourier-transform infrared (FTIR) spectroscopy and by differential scanning calorimetry (DSC).

The FTIR spectra were recorded by means of a Spectrum 100 FTIR spectrometer (PerkinElmer, Waltham, MA, USA) equipped with an attenuated total reflectance accessory (ATR). The scanned wavenumber range was 4000–700 cm^−1^. All the spectra were recorded in environmental conditions (25 °C, 50% RH) with a resolution of 4 cm^−1^, and 8 scans were averaged for each sample. The FTIR identification was performed by using the software Perkin Elmer Spectrum 10 equipped with a proprietary FTIR polymer library database.

DSC analysis was performed on a TA-Q2000 system equipped with an RCS-90 cooling unit (TA Instruments, New Castle, DE, USA). The instrument was calibrated in temperature and energy with pure indium. About 5 mg of each sample were sealed into aluminum pans and subjected to the following temperature program: heating from 25 to 200 °C; cooling from 200 to 25 °C; and heating from 25 to 300 °C. The heating/cooling rate for all the runs was fixed at 10 °C/min.

### 2.2. Analysis of Sorted Plastic Waste

Three different batches of light multi-material plastic wastes were sorted by composition in the semi-automatized sorting plant equipped with NIR detectors of the industrial plant Lavorgna Igiene Urbana srl. The sorting was assisted by a visual inspection of the plant operators. For this work, the PET, PP, PE and PS fractions were collected, washed with deionized water for 1 h at 90 °C and dried in environmental conditions, as described above. Then, the samples were ground at 25 °C and 50% RH with a lab-scale granulator Wanner B08.10 (Wanner Technik GmbH, Wertheim-Reicholzheim, Germany) equipped with sieves with holes of 4 × 4 mm. Selected fragments of the obtained granules were characterized by FTIR spectroscopy using the same equipment and experimental conditions detailed in Section 2.1 for the unsorted plastic waste fractions.

### 2.3. Melt Mixing and Compression Molding of Sorted PP, PS and PE Fractions

The sorted, washed and ground PP, PS and PE granules were processed by melt mixing using a Brabender Plastograph (Brabender GmbH & Co, Duisburg, Germany) internal mixer. The PP and PS fractions were processed at 190 °C for 10 min. The PE fractions were processed at 160 °C for 10 min. For all the systems, the mixing speed was fixed at 60 rpm. After mixing, the processed samples were ground with the above-specified Wanner granulator to obtain granules with a lateral size < 4 mm.

The granules were then used to produce sample plates (size 10 × 10 × 0.1 cm^3^ and 10 × 10 × 0.3 cm^3^) by compression molding using a hot platen press Collin P200 (Collin Lab & Pilot Solutions GmbH, Maitenbeth, Germany). The compression molding temperature was 200 °C for PP and PS, and 160 °C for the PE plates. The samples were compression molded for 10 min, progressively increasing the pressure up to 100 bar. Then, the samples were quickly cooled to room temperature by the water-cooling cassette system of the hot platen press.

The obtained samples were used to perform the elemental analysis, to evaluate the presence of inorganic contaminants. Moreover, the plates were used to evaluate the tensile properties of the PE, PP and PS recycled samples and the flexural properties of the PP and PS recycled samples. The PE samples were not tested in terms of the flexural configuration due to their very low elastic modulus. The elemental analysis was performed by energy dispersive X-ray spectroscopy (EDX). Samples removed from the plates were mounted on aluminum stubs by means of bi-adhesive carbon disks and analyzed using a FEI Quanta 200 (FEI, Eindhoven, The Netherlands) scanning electron microscope (SEM) operating at 30 KV acceleration voltage, equipped with an Oxford Inca LN2 free EDX detector (Oxford Instruments, Abingdon, UK). The analysis was performed on different areas of each sample, and the average amount of each element, as well as its standard deviation, was calculated.

Concerning the evaluation of the processability of the sorted PP, PE and PS fractions, the compounds obtained by melt mixing of the sorted fractions from the 3 different batches were characterized by measuring the melt flow rate (MFR). The MFR (g/10 min) analysis was performed on a Ceast MF20 tester (ITW Test and Measurement Italia S.r.l., Pianezza, TO, Italy). The experimental conditions were as follows: 230 °C/2.16 kg for PP batches, 190 °C/2.16 kg for PE batches, and 200 °C/5 kg for PS batches.

### 2.4. Mechanical Analysis

Tensile tests were performed on PP and PE at room temperature (25 °C) and 50% RH on dumb-bell specimens (4 mm^2^ cross-section, 1 mm thickness, 25 mm gauge length) at a cross-head speed of 10 mm/min by using a 5564 Instron mechanical testing instrument (Instron, Norwood, MA, USA). The Young’s modulus, yield stress, strain at break values and related standard deviation values were calculated as the average values over at least 5 tested samples.

Flexural tests were carried out on the PP and PS samples at room temperature (25 °C) and 50% RH by using an Instron 4505 mechanical testing instrument (Instron, Norwood, MA, USA). The test span was 48 mm and the cross-head speed was 2 mm/min. The flexural modulus, flexural strength and strain at yield (for PP) and at break (PS) and the related standard deviation values were calculated on un-notched samples (60 mm long, 10.0 mm wide, 3.0 mm thick) over at least 5 tested samples.

The results were expressed as average values and the standard deviation values were calculated.

### 2.5. Preparation of Compounds with Coupling Agents and Characterization

Compounds based on recycled PP and PE containing suitable compatibilizers or coupling agents able to possibly promote better adhesion between the recycled polymer phases and the contaminants present in the waste fractions were prepared. The following materials were used as coupling agents:

- Commercial PP grafted with maleic anhydride (PPMA), trade name KA 805, containing approximately 1 wt% of grafted meleic anhydride, kindly supplied by Basell Polyolefins spa (Ferrara, Italy).
- Commercial PE grafted with maleic anhydride (PEMA), trade name Compoline CO/LL, with a grafted maleic anhydride content of 1.4 wt%, kindly supplied by Auser Polimeri srl (Lucca, Italy).

Recycled PP and PE, selected from batch 2 since it was the one with the higher compositional variability, were, respectively, compounded with PPMA and PEMA with the above specified Brabender Plastograph internal mixer, setting the same temperature and mixing time used for the neat PP and PE, respectively. The PP/PPMA weight ratios were 95/5 (PP2_5PPMA) and 90/10 (PP2_10PPMA). Similarly, the PE/PEMA weight ratios were 95/5 (PE2_5PEMA) and 90/10 (PE2_10PEMA).

On these compounds, the MFR was measured as previously specified for the neat recycled PP and PE. Furthermore, the compounds were compression molded, realizing plates suitable for mechanical analysis. Tensile tests were carried out on both the PP/PPMA- and PE/PEMA-based compounds using the same experimental conditions as specified for the corresponding systems prepared without coupling agents.

## 3. Results and Discussion

### 3.1. Compositional Analysis of Unsorted Plastic Waste

A combined FTIR/DSC analysis was used to investigate the composition of the unsorted plastic waste collected at the industrial plant. The results are summarized in the following Table 1, Table 2 and Table 3 for the three different batches analyzed.

**Figure 1:**
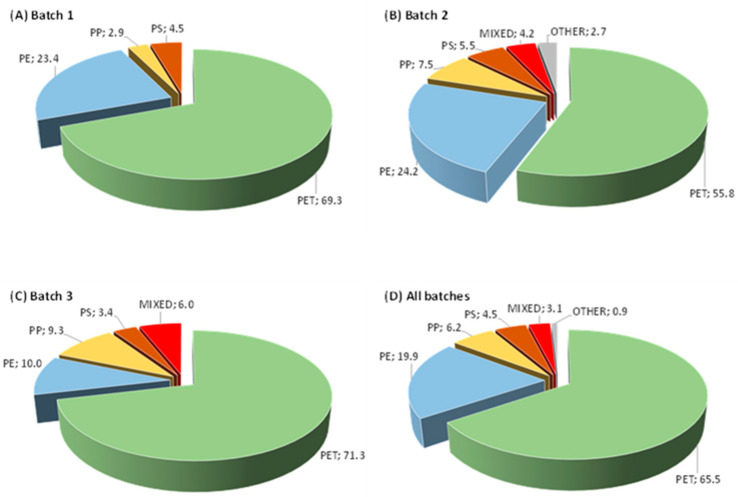
Results of the materials’ identification (wt%) in the different multi-material waste batches (**A**–**C**) and average results obtained for all the batches (**D**).

As shown, the analysis of a total of 120 samples from three different batches of unsorted multi-material waste allowed us to uniquely evaluate the composition of all the samples. The results obtained for the different batches and the total items investigated are summarized in Figure 1. As shown, for all the investigated batches, PET is the predominant component, whose amount in the three different batches ranges from 55.8 to 71.3 wt% (average value, shown in Figure 1D, over all the batches 65.5 wt%), followed by PE, with a highly variable amount, ranging from 10.0 to 24.2 wt % (average value over all the batches 19.9 wt%), PP, with amounts ranging from 2.9 to 9.3 wt% (average value 6.2 wt%), and PS, with amounts ranging from 3.4 to 5.5 wt% (average value 4.5 wt%). Waste items with a mixed composition were also found, with the corresponding amounts ranging from 0 to 6.0 wt% (average value 3.1 wt%), while other materials constituted less than 1 wt% of the total amount of wastes.

### 3.2. Sorting and Analysis of Sorted Plastic Wastes

An industrial sorting of the multi-material wastes was performed on three different batches in an industrial plant, as detailed in the experimental section. By the industrial sorting process, for each batch, four different fractions were recovered: PET, PP, PE, and PS. Then, each separated fraction was collected, washed with deionized water for 1 h at 90 °C, dried in environmental conditions, and ground with a lab-scale granulator, obtaining granules with a lateral size < 4 mm.

For each batch and homogeneous fraction, 100 granules were randomly selected and characterized by FTIR spectroscopy, identifying their composition by software matching with a polymer FTIR database. The results are reported in Table 4. Thus, the FTIR characterization confirmed the effectiveness of the sorting process, revealing the presence of cellulose contaminants only for the PS fraction of batch 2 (PS2).

### 3.3. Melt Mixing and Compression Molding of the PP, PE and PS Polymer Fractions

In order to evaluate the recyclability of the sorted plastic wastes, the sorted fractions constituted by PP, PE and PS were processed by melt mixing, as detailed in the experimental section. For this activity, the PET fractions were excluded because PET, as detailed in Table 1, Table 2 and Table 3, mainly derives from drinking bottle, and thus can be considered a plastic fraction suitable for a mechanical recycling process without significant problems due to its inhomogeneous composition.

#### 3.3.1. Elemental Analysis of PP, PE and PS Polymer Fractions

As detailed in the experimental section, compression molded plates were produced and used to evaluate the presence of inorganic contaminants by EDX spectroscopy. The results are reported in Table 5. As shown, EDX analysis revealed the presence of relatively small amounts of inorganic materials, mainly constituted by elements such as titanium, calcium, silicon and aluminum, often present in common fillers used in the formulation of polymeric compounds. In a few cases, iron (PP2) and chlorine (PS3) were also detected in low amounts, probably as a consequence of external impurities contaminating the materials during the waste collection and transport, which the washing process was unable to remove. In general, the results reported in Table 5 evidenced low amounts of inorganic contaminants that reach values higher than 1 wt% only for calcium.

Although in the analyzed batches the amount of contaminants is very low, the identification and quantification of contaminants is a mandatory step before planning any mechanical recycling process to exclude the presence of harmful and or toxic substances in the recycled materials.

#### 3.3.2. Melt Flow Rate

Before compression molding, recycled compounds prepared by melt mixing and grinding were analyzed by measuring their MFR. The results are reported in Table 6.

The PP samples showed MFR values at 230 °C/2.16 kg ranging between about 31 g/10 min (batch 1) and 15 g/10 min (batch 3), with an average MFR value over the three different batches of 22.5 ± 8.1 g/10. These values are compatible with the MFR values of virgin PP products, although the variability recorded among the different batches suggests some caution in the definition of the processing conditions for recycling.

In detail, the recorded MFR values for the recycled PP batches are compatible with those of commercial virgin PP materials, such as the following:

- Sabic PP 48M40, MFR 15 g/10 min @ 230 °C/2.16 kg, injection-molding grade, used for crates, boxes and rigid packaging.
- LyondellBasell Moplen HP483R, MFR 27 g/10 min @ 230 °C/2.16 kg, injection-molding grade, used for caps and closures, furniture, and household articles.
- Borealis RF365MO, MFR 20 g/10 min @ 230 °C/2.16 kg, used for closures and thin wall containers.

Similarly, the mixed PE samples show MFR values at 190 °C/2.16 kg ranging between about 0.8 g/10 (batch 2) and 1.8 g/10 min (batch 3), with an average and standard deviation MFR value over the three different batches of 1.2 ± 0.5 g/10. These MFR values are compatible with those of some commercially available virgin PE, such as the following:

- Sabic LDPE 2801H0W, MFR 0.55 g/10 min @ 190 °C/2.16 kg, used for shrink films and packaging films.
- LyondellBasell Lupolen 3020F, MFR 0.90 g/10 min @ 190 °C/2.16 kg, used for bags and pouches, food packaging films, and lamination films.
- Dow DOWLEX 2049, MFR 1.0 g/10 min @ 190 °C/2.16 kg, used for food packaging.

Indeed, although some commercial grades of PE for injection molding have significantly higher MFI values, the analyzed waste PE batches contain high amounts of end-of-life packaging films, which are usually produced from PE grades with relatively low MFR.

Finally, the mixed PS samples show MFR values at 200 °C/5 kg ranging between about 6 g/10 min (batches 1 and 3) and 8 g/10 min (batch 2), with an average and standard deviation MFR value over the three different batches of 7.0 ± 1.1 g/10. Also in this case, the recorded MFR values of the recycled PS are compatible with those of virgin PS products, such as the following:

- Ineos Styrolution PS-148G, MFR 6 g/10 min @ 200 °C/5 kg, general purpose polystyrene.
- Sabic PS 155 PS, MFR 7 g/10 min @ 200 °C/5 kg, general purpose polystyrene.

Thus, the overall analyses of the composition, the presence of inorganic contaminants and the MFR of the PP, PE and PS sorted batches evidence the good processability of the recycled products.

### 3.4. Mechanical Analysis of PE, PP and PS Compounds

As detailed in the experimental section, PP, PE and PS plates realized by compression molding were analyzed to evaluate their mechanical properties. In particular, tensile tests were performed on the PP, PE and PS samples, whereas flexural tests were performed on the PP and PS samples. The results are reported in Table 7.

As illustrated in Table 7, irrespective of the analyzed batches, the PP samples show good and highly reproducible Young’s modulus values, ranging from 780 to 860 MPa, and tensile strength (17.1 to 17.8 MPa), with relatively low strain at break values, in all cases close to 5%. Flexural analysis confirmed the good reproducibility of the properties in the three investigated batches, with flexural modulus values in the 525–540 MPa range, stress at yield between 22.5 and 25.5 MPa and strain at yield values between 8.1 and 8.9%.

Nevertheless, comparing the recorded properties with those of commercial virgin PP homopolymer, we can note that both the modulus of the recycled PP batches and the tensile strength are significantly lower. For instance, the homopolymer PP mentioned in the previous section (Sabic PP 48M40, LyondellBasell Moplen HP483R, Borealis RF365MO) show a tensile modulus in the range of 1150–1400 MPa and a tensile yield strength in the range of 26–32 MPa. The low mechanical parameters recorded on our recycled PP can be compared with high-impact modified PP materials (e.g., SABIC PP 90910, elastomer modified, with elastic modulus 850 MPa and yield strength 18 MPa), but without the benefits induced by the modification.

Results with good reproducibility in the three investigate batches were also shown by tensile tests of the PE fractions, with Young’s modulus values in the range of 585–640 MPa, stress at yield between 19.2 and 19.6 MPa and strain at break close to 700%. The high ductility of PE was thus preserved in the recycled materials, despite the presence of inorganic additives and contaminants.

For the recycled PE, by comparing the recorded mechanical properties with those shown by commercial virgin PE, we can note interesting similarities.

For instance, commercial LDPE materials such as the already mentioned Sabic LDPE 2801H0W, LyondellBasell Lupolen 3020F, and Dow DOWLEX 2049 show a tensile modulus in the range of 300–340 MPa, a stress at yield in the range of 12–15 MPa, with an ultimate elongation > 500%. Being a mixture of different low- and high-density PE grades, our recycled PE shows a ductility similar to most commercial LDPE, with somewhat higher stiffness and strength. The mechanical response is close to that of (partly) recycled commercial PE such as Versalis Revive COM70HF1 (70% recycled content, elastic modulus 650 MPa, yield strength not declared), or LyondellBasell CirculenRecover HD45U06 (40% recycled content, elastic modulus 850 MPa, yield strength 24 MPa).

Interesting results were also obtained on the PS fractions, also showing low variability in the properties of the three analyzed batches. Indeed, tensile tests revealed a Young’s modulus between 1400 and 1455 MPa, a tensile strength between 20.2 and 21.1 MPa and strain at break values close to 1.5% due to the brittle nature of the polymer. Similar results were obtained by flexural tests of PS, with a flexural modulus value close to 1650 MPa for all the PS1, PS2 and PS3 batches, flexural strength between 27.1 and 28.8 MPa and strain at break values close to 3%.

For the recycled PS, by comparing the recorded mechanical properties with those shown by a commercial virgin PS, we can note that both the flexural modulus of the recycled PS batches and the flexural strength are significantly lower. For instance, the PS homopolymer PS 155 (Sabic) shows a flexural strength of 66 MPa and a flexural modulus of about 3600 MPa. Also, in this case, the properties of the recycled PS are better compared to the modified PS grades, like the high impact Ineos Styrolution PS 476L, flexural modulus of 1950 MPa, and flexural strength of 40 MPa.

In general, the variability of the mechanical properties of the same recycled polymers over different batches of sorted wastes was very low, with deviations in all cases similar or lower than those recorded amongst samples belonging to the same batch. For the PP and PS recycled materials, the recorded mechanical properties are significantly lower than those expected for a virgin homopolymer. This finding is not new in comparison to the available literature data. In general, the properties of recycled polyolefin blends are much worse than those of virgin polymers [49] and different approaches based on the additivation of sorted plastic fractions with virgin fractions [50,51], the implementation of close recycled loops to prevent contamination [52] or the use of performance improvers [53] are needed to obtain recycled compounds with properties more similar to those of virgin materials.

Therefore, the results confirmed that the recycling of PP, PE and PS polymer fractions is able to produce recycled compounds with highly reproducible properties, which are then suitable for the production of recycled compounds, although effective strategies to improve the properties of recycled compounds must be considered, such as mixing them with virgin polymer fractions to improve their properties and making them suitable for products with good commercial value.

### 3.5. Property Improvement of Recycled PP and PE Fractions

In order to evaluate the possibility of further improving the mechanical properties of PP and PE recycled materials, sorted and grinded granules were additivated with coupling agents in order to possibly promote better adhesion between the recycled polymer phases and the contaminants present in the waste fractions.

For this reason, PPMA and PEMA were, respectively, added to the PP and the PE granules of the second batch at 5 and 10 wt% loading (with respect to the amount of PP and PE, respectively). The obtained samples were coded PP2_5PPMA, PP2_10PPMA, PE2_5PEMA and PE2_10PEMA.

First, MFR tests were performed on the compounds containing the selected coupling agents.

For PP, by the addition of PPMA, the MFR (230 °C/2.16 kg) only slightly increased, from 20.61 g/10 min (PP2) to 21.12 g/10 min at 5 wt% PPMA content (PP2_5PPMA) and to 21.54 g/10 min at 10 wt% PPMA content (PP2_10PPMA). A negligible increase in the MFR (190 °C/2.16 kg) was instead recorded for PE, whose MFR changed from 0.78 g/10 min (PE2) to 0.84 at 5 wt% PEMA content (PE2_5PEMA) and to 0.81 g/10 min at 10 wt% PEMA loading (PE2_10PEMA).

The melt-mixed and compression-molded samples were then analyzed by tensile tests. The results are reported in Table 8, where the tensile properties of PP2 and PE2 are also repeated from Table 7 for comparison. As shown, the addition of 5 wt% of PPMA to the recycled PP compound mainly induced an appreciable increase in the tensile strength, which passed from 17.1 MPa for PP2 to 21.6 MPa for PP2_5PPMA. The addition of 5 wt% of PPMA also increased the strain at break of the PP, while it had a non-appreciable effect on the Young’s modulus. Further addition of PPMA (10 wt% for the sample PP2_10PPMA) did not induce a further improvement of the mechanical properties of the recycled PP, confirming that, due to the low amount of impurities, 5 wt% is the optimal amount to promote an improvement of the mechanical properties of the recycled PP fraction.

Concerning the PE fraction, instead, it is to be noted that only at 5 wt% of PEMA loading was a slight increase of the tensile strength of the PE2 recorded (from 19.4 to 21.0 MPa), while the Young’s modulus and the strain at break were unaffected by the PEMA addition. Also, for PE, the addition of 10 wt% of PEMA did not induce an appreciable improvement of the tensile properties of the recycled fraction.

Therefore, in order to promote an improvement of the properties of the recycled PP and PE fractions, a strategy based on the addition of low amounts of maleated PP or PE (up to 5 wt%) able to improve the adhesion between the recycled polymer phase and the inorganic contaminants can be applied. Nevertheless, when the amount of contaminants is low, such as in the present case study, the use of maleated agents does not induce dramatic improvements of the properties and thus a decision about their addition must be taken after performing a detailed cost–benefit and environmental impact analysis. Nevertheless, it must be underlined that previous studies have evidenced that whereas the use of performance improvers significantly affects the economic impact of a recycling process, in mechanical recycling, compatibilizers and other additives added at the step of compounding or re-extrusion do not have any significant impact on the overall LCA of the recycling process [54,55].

### 3.6. Classification of Sorted Polymers

The creation of a database, potentially exploitable with the help of AI, containing specific information regarding the processes involving the recycling of a polymer can be an effective way to efficiently predict the performances of recycled materials. With this aim, compounding industries would benefit from a comprehensive classification method for sorted and compounded recycled fractions that is able to quickly evidence the main properties of the recycled compounds.

On the basis of the tests carried out and the results previously discussed, a classification system was designed based on the MFR, the Young’s modulus and the tensile strength of PP and PE, and on the flexural modulus and the flexural strength of PS. In addition, for the PE fraction, a final code will define the elongation at break.

In particular, based on the analysis of virgin and recycled materials, the classes of materials specified in Table 9 were hypothesized for PP, PE and PS. For each property, a progressive subcode was defined, with a number that progressively increases with increasing the specified property.

For instance, considering the example of PP, the subcodes for the MFR are R0, R1, R2, R3, R4 and R5 for MRF values that progressively increase from values < 5 to values > 40 g/10 min in standard measuring conditions suitable for testing PP compounds. Similarly, for the Young’s modulus, the subcodes vary from M0 to M5 for Young’s modulus values increasing from <500 MPa to values > 2000 MPa. The same approach (see Table 9) was used for the tensile strength, with subcodes S0–S5 for materials showing tensile strength values increasing from values < 10 MPa to values > 30 MPa. The same approach was used for the PS compounds, as detailed in Table 9, considering for PS the MFR values and flexural modulus and strength. For PE, a further subcode (E0–E5) was hypothesized, which takes into account the elongation at break, a very relevant parameter for this ductile polymer. Although the elongation at break is very relevant for PP, too, in the case of recycled PP fractions, they always contain relevant amounts of inorganic additives and pigments, such as calcium carbonate, talc, and titanium dioxide, and these substances strongly affect the elongation at break of recycled PP compounds. Indeed, in our case, the elongation at break of the recycled PP compounds obtained from different batches never overcame 5.9% (see Table 7), and also for recycled compounds containing coupling agents, the maximum strain at break recorded was 8.7% (Table 8). For the limited range of strain at break values that can be obtained for the recycled PP batches, we excluded this parameter from those identified to design a classification method for recycled polymers.

Therefore, for each PP or PS fraction, a compound will be characterized with a classification code:

Polymer_Rx_My_Sz, where:

Polymer indicates the type of polymer (PP or PS);

Rx, where x is an integer number from 0 to 5, which indicates the MFR range under the typical experimental conditions, respectively, used for PP or PS specified in Table 9;

My, where y is an integer number from 0 to 5, which indicates the range of Young’s modulus values for PP or the range of flexural modulus values for PS specified in Table 9;

Sz, where z is an integer number from 0 to 5, which indicates the range of tensile stress at yield for PP or flexural stress at break for PS specified in Table 9.

For PE, instead, a compound will be characterized by the following classification code:

PE_Rx_My_Sz_Ew, where:

Rx, My and Sz are the same subcodes used for PP, with the range properly defined on the basis of typical properties of PE, as specified in Table 9;

Ew, where w is an integer number from 0 to 5, which indicates the elongation at break range of PE during tensile tests, as also detailed in Table 9.

In addition to the previous subcodes Rx, My, Sz and Ew, a further subcode can be used, C%, to indicate the addition of a performance improver, such as a coupling agent, with the % value indicating the wt% amount of this agent in the compound.

Thus, for PP and PS, the classification codes would become:

Polymer(C%)_Rx_My_Sz

And for PE:

PE(C%)_Rx_My_Sz_Ew

Thus, considering the recycled compounds reported in the previous sections and the results obtained by their MFR and mechanical characterization, each compound can be classified as reported in Table 10.

As shown in Table 10, an overlap between the subcodes related to the MFI and mechanical properties can be observed for certain grades of recycled PP and recycled PE, but this does not affect the validity of the classification, as the first subcode for each material clearly reports the type of polymer. Thus, the proposed classification system is able to clearly show salient data in the code to quickly visualize the processability and the main mechanical properties of the recycled samples, including the eventual amount of performance improvers.

## 4. Conclusions

In this work, we report the results obtained by a case study investigation of different batches of multi-material post-consume plastic wastes collected in an industrial sorting plant in Italy. The batches were characterized by compositional analysis of homogenous plastic fractions collected through industrial sorting, evaluation of their processability through MFR measurements and assessment of the performances of recycled compounds obtained from the different batches.

The sorting allowed us to obtain PP, PE and PS fractions. The accuracy of the sorting was proved through combined spectrometric and calorimetric analysis, revealing the presence of very small amounts of polymeric contaminants in each sorted fraction. The sorted fractions were then ground and processed by melt mixing. The MFR of the recycled compounds revealed low variability amongst different batches of each sorted fraction and MFR values comparable to those of commercial virgin analogous polymers. The recycled compounds were then processed into plates by compression molding and mechanically characterized by tensile and flexural tests. The results showed that the recycled PP and PS compounds have mechanical properties appreciably lower than those shown by commercial virgin analogous, while the recycled PE fraction shows satisfactory modulus, tensile strength and ductility in comparison to virgin PE.

Moreover, we investigated the effectiveness of performance improvers in terms of the properties of the recycled PP and PE compounds, realized by additivation during the reprocessing of the recycled waste fraction with maleated PP and PE coupling agents, respectively. The use of the coupling agent induced an improvement of the tensile strength and elongation at break of PP, whereas it had only a slight effect on the recycled PE batches, demonstrating that the effectiveness of performance improvers depends on the composition and contamination level and a decision about their use needs a detailed cost–benefit analysis.

Overall, the results demonstrate that by mechanical recycling of different batches coming from the same collection and sorting plant, recycled products with low variability in terms of the processability and mechanical properties can be obtained. Recycled PE compounds have also good performances, while recycled PP and PS compounds have mechanical properties appreciably lower than those shown by commercial virgin analogous.

Moreover, when the amount of contaminants in the sorted plastic fractions is low, such as in the present case study, the use of maleated agents does not induce dramatic improvements of the properties and thus a decision about their addition must be taken after performing a detailed cost–benefit and environmental impact analysis.

Finally, a classification system for the recycled products has been created based on the MFR and mechanical results obtained by the characterization of the recycled compounds, with the aim of building a database for AI systems, paving the way to predict the characteristics of recycled polymers obtained from urban solid waste.

All these findings are of great interest in defining effective industrial mechanical recycling strategies for multi-material plastic wastes. Nevertheless, the results obtained by the reprocessing and characterization of recycled fractions as well as the application of the proposed classification method should be validated on a large number of sorted and recycled waste batches, also coming from plants located in different geographic areas, possibly extending the study to the analysis of the effect of the sorting efficiency and the presence of contaminants in the sorted plastic fractions on the economic and environmental impacts of the mechanical recycling processes.

## Figures and Tables

**Table 1:** Results of the identification of the unsorted waste materials (batch 1).

Item #	Description	Weight (g)	Tm or Tg * (°C)	Automatic FTIR Identification (Matching %)	Combined DSC/FTIR Identification
1.1	Detergent container	54.2	140 (Tm)	PE (>98%)	PE
1.2	Detergent container	38.4	143 (Tm)	PE (>98%)	PE
1.3	Detergent container	44.4	137 (Tm)	PE (>98%)	PE
1.4	Detergent container	32.4	139 (Tm)	PE (>98%)	PE
1.5	Shopper	2.6	108 (Tm)	PE (>97%)	PE
1.6	Packaging film	1.4	121 (Tm)	PE (>98%)	PE
1.7	Shopper	2.1	121 (Tm)	PE (>98%)	PE
1.8	Shopper	2.4	123 (Tm)	PE (>98%)	PE
1.9	Beverage bottle	40.2	247 (Tm)	PET (>99%)	PET
1.10	Beverage bottle	40.9	246 (Tm)	PET (>99%)	PET
1.11	Beverage bottle	40.1	246 (Tm)	PET (>99%)	PET
1.12	Beverage bottle	42.3	248 (Tm)	PET (>99%)	PET
1.13	Oil bottle	28.8	245 (Tm)	PET (>99%)	PET
1.14	Food tray	8.5	245 (Tm)	PET (>98%)	PET
1.15	Food tray	6.5	245 (Tm)	PET (>98%)	PET
1.16	Food tray	6.2	244 (Tm)	PET (>98%)	PET
1.17	Beverage bottle	20.8	246 (Tm)	PET (>99%)	PET
1.18	Beverage bottle	40.3	247 (Tm)	PET (>99%)	PET
1.19	Beverage bottle	41.8	248 (Tm)	PET (>99%)	PET
1.20	Beverage bottle	40.8	245 (Tm)	PET (>99%)	PET
1.21	Beverage bottle	40.2	247 (Tm)	PET (>99%)	PET
1.22	Beverage bottle	10.1	247 (Tm)	PET (>99%)	PET
1.23	Beverage bottle	10.6	246 (Tm)	PET (>99%)	PET
1.24	Beverage bottle	9.4	248 (Tm)	PET (>99%)	PET
1.25	Beverage bottle	40.1	248 (Tm)	PET (>99%)	PET
1.26	Beverage bottle	9.1	247 (Tm)	PET (>99%)	PET
1.27	Beverage bottle	10.4	246 (Tm)	PET (>99%)	PET
1.28	Beverage bottle	25.3	246 (Tm)	PET (>99%)	PET
1.29	Single-use plate	3.5	162 (Tm)	PP (>98%)	PP
1.30	Packaging film	1.4	160 (Tm)	PP (>98%)	PP
1.31	Food tray	6.4	159 (Tm)	PP (>98%)	PP
1.32	Packaging film	2.4	157 (Tm)	PP (>98%)	PP
1.33	Packaging film	1.6	158 (Tm)	PP (>97%)	PP
1.34	Shopper	3.1	160 (Tm)	PP (>98%)	PP
1.35	Single-use plate	3.4	161 (Tm)	PP (>99%)	PP
1.36	Plant container	12.5	99 (Tg)	PS (>95%)	PS
1.37	Packaging net	3.2	97 (Tg)	PS (>98%)	PS
1.38	Expanded packaging	9.4	101 (Tg)	PS (>98%)	PS
1.39	Packaging film	6.8	110 (Tg)	PS (>97%)	PS
1.40	Packaging item	6.2	96 (Tg)	PS (>98%)	PS

* Melting temperature (Tm), glass transition temperature (Tg).

**Table 2:** Results of the identification of the unsorted waste materials (batch 2).

Item #	Description	Weight (g)	Tm or Tg * (°C)	Automatic FTIR Identification (Matching %)	Combined DSC/FTIR Identification
2.1	Detergent container	38.4	130 (Tm)	PE (>97%)	PE
2.2	Detergent container	28.7	141 (Tm)	PE (>98%)	PE
2.3	Detergent container	19.4	130 (Tm)	PE (>99%)	PE
2.4	Milk bottle	12.2	135 (Tm)	PE (>99%)	PE
2.5	Detergent container	18.2	139 (Tm)	PE (>99%)	PE
2.6	Packaging	6.2	120 (Tm)	PE (>98%)	PE
2.7	Shopper	6.2	120 (Tm)	PE (>98%)	PE
2.8	Flexible packaging	8.4	119 (Tm)	PE (>95)	PE
2.9	Shopper	4.2	125 (Tm)	PE (>96%)	PE
2.10	Flexible packaging	3.2	114 (Tm)	PE (>99)	PE
2.11	Flexible packaging	5.6	116 (Tm)	PE (>97)	PE
2.12	Food tray	6.2	123 (Tm) 150 (Tm)	PE (>97%)/ PP (>98)	PE/PP
2.13	Flexible packaging	5.6	109 (Tm) 177 (Tm)	PE (>98%)/ PP (>98)	PE/PP
2.14	Pot for plants	8.2	122 (Tm) 160 (Tm)	PE (>97%)/ PP (>96)	PE/PP
2.15	Compact disk	14.8	249 (Tm)	PC (>95%)	PC
2.16	Toy	2.2	53 (Tm)	PCL (>88%)	PCL
2.17	Beverage bottle	30.4	249 (Tm)	PET (>99%)	PET
2.18	Beverage bottle	18.2	246 (Tm)	PET (>98%)	PET
2.19	Beverage bottle	28.2	251 (Tm)	PET (>99%)	PET
2.20	Beverage bottle	32.4	250 (Tm)	PET (>99%)	PET
2.21	Beverage bottle	29.3	251 (Tm)	PET (>99%)	PET
2.22	Beverage bottle	18.9	249 (Tm)	PET (>98%)	PET
2.23	Beverage bottle	10.2	253 (Tm)	PET (>99%)	PET
2.24	Beverage bottle	9.4	255 (Tm)	PET (>99%)	PET
2.25	Milk bottle	8.4	248 (Tm)	PET (>99%)	PET
2.26	Beverage bottle	28.7	244 (Tm)	PET (>98%)	PET
2.27	Beverage bottle	23.4	245 (Tm)	PET (>99%)	PET
2.28	Beverage bottle	10.3	255 (Tm)	PET (>99%)	PET
2.29	Case for liquids	9.1	165 (Tm)	PP (>97%)	PP
2.30	Packaging for fruit	12.4	162 (Tm)	PP (>97%)	PP
2.31	Food tray	10.5	163 (Tm)	PP (>97%)	PP
2.32	Flexible packaging	6.3	162 (Tm)	PP (>99)	PP
2.33	Single-use glass	2.1	155 (Tm)	PP (>98%)	PP
2.34	Container for liquids	6.2	163 (Tm)	PP (>99%)	PP
2.35	Flexible packaging	6.2	159 (Tm) 252 (Tm)	PP (>97%)/ PET (>98%)	PP/PET
2.36	Expanded packaging	7.2	90 (Tg)	PS (>97%)	PS
2.37	Food tray	9.4	82 (Tg)	PS (>98%)	PS
2.38	Expanded packaging	4.2	85 (Tg)	PS (>98%)	PS
2.39	Expanded packaging	9.2	80 (Tg)	PS (>98%)	PS
2.40	Food tray	4.3	82 (Tg)	PS (>97%)	PS

* Melting temperature (Tm), glass transition temperature (Tg).

**Table 3:** Results of the identification of the unsorted waste materials (batch 3).

Item #	Description	Weight (g)	Tm or Tg * (°C)	Automatic FTIR Identification (Matching %)	Combined DSC/FTIR Identification
3.1	Detergent container	14.1	139 (Tm)	PE (>99%)	PE
3.2	Detergent container	9.9	133 (Tm) 185 (Tm)	PE (>97%)/ PP (>92%)	PE/PP
3.3	Flexible packaging	2.4	111 (Tm)	PE (>98%)	PE
3.4	Flexible packaging	4.2	110 (Tm)	PE (>99%)	PE
3.5	Flexible packaging	3.8	116 (Tm)	PE (>99%)	PE
3.6	Flexible packaging	4.5	114 (Tm)	PE (>98%)	PE
3.7	Packaging film	4.5	120 (Tm)	PE (>99%)	PE
3.8	Packaging film	3.8	107 (Tm)	PE (>99%)	PE
3.9	Soap container	9.1	137 (Tm)	PE (>98%)	PE
3.10	Packaging container	8.2	122 (Tm)	PE (>98%)	PE
3.11	Shopper	6.2	110 (Tm) 123 (Tm)	PE (>94%)	PE. PE
3.12	Flexible packaging	7.5	114 (Tm) 126 (Tm) 254 (Tm)	PE (>95%)/ PET (>98%)	PE. PE. PET
3.13	Flexible packaging	6.7	101 (Tm) 153 (Tm)	PE (>98%)/ PP (>99%)	PE. PP
3.14	Packaging net	2.4	117 (Tm) 162 (Tm)	PE (>97%)/ PP (>94%)	PE. PP
3.15	Beverage bottle	29.4	245 (Tm)	PET (>99%)	PET
3.16	Beverage bottle	18.4	246 (Tm)	PET (>98%)	PET
3.17	Beverage bottle	42.3	249 (Tm)	PET (>99%)	PET
3.18	Beverage bottle	19.4	251 (Tm)	PET (>98%)	PET
3.19	Beverage bottle	32.3	253 (Tm)	PET (>98%)	PET
3.20	Beverage bottle	19.7	243 (Tm)	PET (>99%)	PET
3.21	Beverage bottle	12.1	240 (Tm)	PET (>99%)	PET
3.22	Milk bottle	9.4	244 (Tm)	PET (>99%)	PET
3.23	Beverage bottle	12.4	245 (Tm)	PET (>99%)	PET
3.24	Beverage bottle	11.3	249 (Tm)	PET (>99%)	PET
3.25	Single use gloves	12.2	254 (Tm)	PET (>97%)	PET
3.26	Beverage bottle	10.4	250 (Tm)	PET (>99%)	PET
3.27	Beverage bottle	27.7	250 (Tm)	PET (>99%)	PET
3.28	Beverage bottle	34.7	245 (Tm)	PET (>98%)	PET
3.29	Beverage bottle	12.9	248 (Tm)	PET (>98%)	PET
3.30	Beverage bottle	39.2	247 (Tm)	PET (>98%)	PET
3.31	Beverage bottle	29.6	246 (Tm)	PET (>98%)	PET
3.32	Food tray	4.8	155 (Tm)	PP (>97%)	PP
3.33	Office item	6.2	158 (Tm)	PP (>99%)	PP
3.34	Detergent container	28.2	164 (Tm)	PP (>98%)	PP
3.35	Shopper	3.2	160 (Tm)	PP (>99%)	PP
3.36	Container	8.2	161 (Tm)	PP (>99%)	PP
3.37	Toy	4.6	96 (Tg)	PS (>99%)	PS
3.38	Single-use plate	2.4	85 (Tg)	PS (>97%)	PS
3.39	Expanded packaging	7.2	87 (Tg)	PS (>97%)	PS
3.40	Packaging tray	4.3	86 (Tg)	PS (>94%)	PS

* Melting temperature (Tm), glass transition temperature (Tg).

**Table 4:** Results of the identification of granules obtained by the grinding of the sorted plastic wastes.

Batch	Fraction	Identification Results by FTIR (% of Analyzed Granules)
1	PET1	PET: 100%
2	PET2	PET: 100%
3	PET3	PET: 100%
1	PP1	PP: 100%
2	PP2	PP: 100%
3	PP3	PP: 100%
1	PE1	PE: 100%
2	PE2	PE: 100%
3	PE3	PE: 100%
1	PS1	PS: 100%
2	PS2	PS: 98%; cellulose: 2%
3	PS3	PS: 100%

**Table 5:** Results of the elemental analysis of the PP, PE and PS sorted fractions in different batches.

Batch	Fraction	Elemental Composition (wt%)							
		C	O	Al	Si	Cl	Ca	Ti	Fe
1	PP1	94.36 ± 0.15	4.76 ± 0.23	0.03 ± 0.02	0.13 ± 0.01	-	0.42 ± 0.07	0.33 ± 0.01	-
1	PE1	93.17 ± 0.38	6.13 ± 0.41	0.08 ± 0.02	0.08 ± 0.01	-	0.15 ± 0.01	0.41 ± 0.01	-
1	PS1	90.20 ± 0.22	7.20 ± 0.16	-	0.10 ± 0.03	-	0.98 ± 0.08	1.28 ± 0.14	
2	PP2	92.01 ± 0.28	6.06 ± 0.11	0.11 ± 0.02	0.32 ± 0.01	-	1.05 ± 0.15	0.41 ± 0.02	0.07 ± 0.03
2	PE2	93.88 ± 0.32	5.14 ± 0.35	0.06 ± 0.03	0.07 ± 0.01	-	0.24 ± 0.01	0.63 ± 0.02	-
2	PS2	90.25 ± 0.45	7.57 ± 0.49	-	0.19 ± 0.08	-	1.15 ± 0.01	0.83 ± 0.03	-
3	PP3	94.72 ± 0.64	3.72 ± 0.42	0.06 ± 0.02	0.35 ± 0.01	-	0.79 ± 0.20	0.38 ± 0.01	-
3	PE3	93.20 ± 1.76	5.58 ± 1.82	0.11 ± 0.04	0.18 ± 0.05	-	0.81 ± 0.06	0.14 ± 0.01	-
3	PS3	90.60 ± 0.12	7.00 ± 0.36	-	0.09 ± 0.02	0.22 ± 0.04	0.92 ± 0.06	1.17 ± 0.11	-

**Table 6:** Results of the MFR analysis of the PP, PE and PS sorted fractions from different batches.

Batch	Fraction	Test Conditions	MFR (g/10 min)
1	PP1	230 °C/2.16 kg	31.36
2	PP2	230 °C/2.16 kg	20.61
3	PP3	230 °C/2.16 kg	15.41
1	PE1	190 °C/2.16 kg	0.96
2	PE2	190 °C/2.16 kg	0.78
3	PE3	190 °C/2.16 kg	1.79
1	PS1	200 °C/5 kg	6.34
2	PS2	200 °C/5 kg	8.35
3	PS3	200 °C/5 kg	6.46

**Table 7:** Results of the mechanical analysis of the PP, PE and PS recycled samples.

**Tensile Tests**			
Sample	Young’s modulus (MPa)	Stress at yield (MPa)	Strain at break (%)
PP1	855 ± 15	17.8 ± 1.8	5.8 ± 1.9
PP2	860 ± 25	17.1 ± 1.4	5.6 ± 0.9
PP3	780 ± 35	17.6 ± 1.8	5.9 ± 0.4
**PP average over 3 batches**	**832 ± 45**	**17.5 ± 0.4**	**5.8 ± 0.2**
PE1	585 ± 35	19.6 ± 0.6	770 ± 50
PE2	640 ± 45	19.4 ± 0.5	735 ± 80
PE3	595 ± 25	19.2 ± 0.1	680 ± 100
**PE average over 3 batches**	**606 ± 30**	**19.4 ± 0.2**	**728 ± 45**
PS1	1400 ± 120	20.2 ± 2.4	1.4 ± 0.2
PS2	1455 ± 100	21.1 ± 2.2	1.6 ± 0.1
PS3	1410 ± 65	20.8 ± 1.7	1.5 ± 0.2
**PS average over 3 batches**	**1422 ± 21**	**20.7 ± 0.5**	**1.5 ± 0.1**
**Flexural tests**			
Sample	Flexural modulus (MPa)	Stress at yield/break (MPa)	Strain at yield/break (%)
PP1	530 ± 80	24.5 ± 3.1 *	8.1 ± 0.3 *
PP2	545 ± 60	25.5 ± 2.8 *	8.6 ± 0.2 *
PP3	525 ± 25	22.5 ± 1.2 *	8.9 ± 0.2 *
**PP average over 3 batches**	**533 ± 10**	24.1 ± 1.5 *	8.5 ± 0.4 *
PS1	1720 ± 110	27.1 ± 1.4 **	3.3 ± 0.1 **
PS2	1650 ± 150	27.4 ± 1.8 **	3.0 ± 0.4 **
PS3	1580 ± 70	28.8 ± 2.4 **	2.9 ± 0.3 **
**PS average over 3 batches**	**1650 ± 70**	27.8 ± 0.9 **	3.1 ± 0.2 **

* yield; ** break. Standard deviations on the PP, PE and PS average values are calculated on the three batches average values, i.e., they are not dependent on the errors on the single batches measurements.

**Table 8:** Results of the mechanical analysis of the PP and PE recycled samples containing coupling agents.

Tensile Tests			
Sample	Young’s Modulus (MPa)	Stress at Yield (MPa)	Strain at Break (%)
PP2_5PPMA	855 ± 45	21.6 ± 0.7	8.5 ± 1.6
PP2_10PPMA	790 ± 45	21.1 ± 1.0	8.7 ± 1.8
PP2	860 ± 25	17.1 ± 1.4	5.6 ± 0.9
PE2_5PEMA	660 ± 45	21.0 ± 0.4	635 ± 105
PE2_10PEMA	650 ± 30	19.3 ± 0.8	570 ± 40
PE2	640 ± 45	19.4 ± 0.5	735 ± 80

**Table 9:** Classification ranges and subcodes for each specific property for PP, PE and PS.

**PP**											
MFR @ 230 °C/2.16 kg (g/10 min)		Sub code		Young’s modulus @ 10 mm/min (MPa)		Sub code		Stress at yield @ 10 mm/min (MPa)		Sub code	
<5		R0		<500		M0		<10		S0	
5–10		R1		500–875		M1		10–15		S1	
10–20		R2		875–1250		M2		15–20		S2	
20–30		R3		1250–1625		M3		20–25		S3	
30–40		R4		1625–2000		M4		25–30		S4	
>40		R5		>2000		M5		>30		S5	
**PE**											
MFR 190 °C/2.16 kg (g/10 min)	Sub code		Young’s modulus @ 10 mm/min (MPa)		Sub code	Stress at yield @ 10 mm/min (MPa)	Sub code		Strain at break @ 10 mm/min (%)		Sub code
<0.3	R0		<400		M0	<15.0	S0		<100		E0
0.3–0.8	R1		400–600		M1	15.0–17.5	S1		100–300		E1
0.8–1.3	R2		600–800		M2	17.5–20.0	S2		300–500		E2
1.3–1.8	R3		800–1000		M3	20.0–22.5	S3		500–700		E3
1.8–2.3	R4		1000–1200		M4	22.5–25.0	S4		700–900		E4
>2.3	R5		>1200		M5	>25	S5		>900		E5
**PS**											
MFR 200 °C/5 kg (g/10 min)		Subcode		Flexural modulus @ 2 mm/min (MPa)		Subcode		Stress at break @ 2 mm/min (MPa)		Subcode	
<4		R0		<1000		M0		<20.0		S0	
4–6		R1		1000–1400		M1		20.0–23.0		S1	
6–8		R2		1400–1800		M2		23.0–26.0		S2	
8–10		R3		1800–2200		M3		26.0–29.0		S3	
10–12		R4		2200–2600		M4		29.0–32.0		S4	
>12		R5		>2600		M5		>32		S5	

**Table 10:** Classification of the recycled compounds.

Batch	Sample	Classification Code
1	PP1	PP_R4_M1_S2
1	PE1	PE_R2_M1_S2_E4
1	PS1	PS_R2_M2_S3
2	PP2	PP_R3_M1_S2
2	PP2_5PPMA	PP(C5)_R3_M1_S3
2	PP2_10PPMA	PP(C10)_R3_M1_S3
2	PE2	PE_R1_M2_S2_E4
2	PE2_5PEMA	PE(C5)_R2_M2_S3_E3
2	PE2_10PEMA	PE(C10)_R2_M2_S2_E3
2	PS2	PS_R3_M2_S3
3	PP3	PP_R2_M1_S2
3	PE3	PE_R3_M1_S2_E3
3	PS3_MIX	PS_R2_M2_S3

## Data Availability

Data supporting the findings of this study are available within the article. Further data are available on request from the corresponding author, RA.

## References

[1] Avella M., Avolio R., Bonadies I., Carfagna C., Errico M.E., Gentile G. (2009). Recycled multilayer cartons as cellulose source in HDPE-based composites: Compatibilization and structure-properties relationships. J. Appl. Polym. Sci..

[2] Piergiovanni L., Limbo S. (2016). Plastic Packaging Materials. Food Packaging Materials.

[3] Evode N., Ahmad Qamar S., Bilal M., Barceló D., Iqbal H.M.N. (2021). Plastic waste and its management strategies for environmental sustainability. Case Stud. Chem. Environ. Eng..

[4] Marsh K., Bugusu B. (2007). Food packaging—Roles, materials, and environmental issues. J. Food Sci..

[5] Guerritore M., Olivieri F., Castaldo R., Avolio R., Cocca M., Errico M.E., Galdi M.R., Carfagna C., Gentile G. (2022). Recyclable-by-design mono-material flexible packaging with high barrier properties realized through graphene hybrid coatings. Resour. Conserv. Recy..

[6] Nayanathara Thathsarani Pilapitiya P.G.C., Ratnayake A.S. (2024). The world of plastic waste: A review. Clean. Mater..

[7] Kibria M.G., Masuk N.I., Safayet R., Nguyen H.Q., Mourshed M. (2023). Plastic Waste: Challenges and Opportunities to Mitigate Pollution and Effective Management. Int. J. Environ. Res..

[8] Koller M., Braunegg G. (2018). Advanced approaches to produce polyhydroxyalkanoate (PHA) biopolyesters in a sustainable and economic fashion. EuroBiotech J..

[9] Lau W.W., Shiran Y., Bailey R.M., Cook E., Stuchtey M.R., Koskella J., Velis C.A., Godfrey L., Boucher J., Murphy M.B. (2020). Evaluating scenarios toward zero plastic pollution. Science.

[10] Rhodes C.J. (2018). Plastic pollution and potential solutions. Sci. Prog..

[11] Jambeck J.R., Geyer R., Wilcox C., Siegler T.R., Perryman M., Andrady A., Narayan R., Law K.L. (2015). Plastic waste inputs from land into the ocean. Science.

[12] Galloway T.S., Lewis C.N. (2016). Marine microplastics spell big problems for future generations. Proc. Natl. Acad. Sci. USA.

[13] Chen Q., Allgeier A., Yin D., Hollert H. (2019). Leaching of endocrine disrupting chemicals from marine microplastics and mesoplastics under common life stress conditions. Environ. Int..

[14] Chamas A., Moon H., Zheng J., Qiu Y., Tabassum T., Jang J.H., Abu-Omar M., Scott S.L., Suh S. (2020). Degradation rates of plastics in the environment. ACS Sustain. Chem. Eng..

[15] Vencato S., Montano S., Saliu F., Coppa S., Becchi A., Liotta I., Valente T., Cocca M., Matiddi M., Camedda A. (2024). Phthalate levels in common sea anemone Actinia equina and Anemonia viridis: A proxy of short-term microplastic interaction?. Mar. Pollut. Bull..

[16] Meng J., Zhang Q., Zheng Y., He G., Shi H. (2024). Plastic waste as the potential carriers of pathogens. Curr. Opin. Food Sci..

[17] Tang Y., Liu Y., Chen Y., Zhang W., Zha J., He S., Yang C., Zhang T., Tang C., Zhang C. (2021). A review: Research progress on microplastic pollutants in aquatic environments. Sci. Total Environ..

[18] Thompson R.C., Olsen Y., Mitchell R.P., Davis A., Rowland S.J., John A.W.G., McGonigle D., Russell A.E. (2004). Lost at sea: Where is all the plastic?. Science.

[19] De Falco F., Gullo M.P., Gentile G., Di Pace E., Cocca M., Gelabert L., Brouta-Agnésa M., Rovira A., Escudero R., Villalba R. (2018). Evaluation of microplastic release caused by textile washing processes of synthetic fabrics. Environ. Pollut..

[20] Eriksen M., Lebreton L.C.M., Carson H.S., Thielm M., Moore C.J. (2024). Plastic pollution in the world’s oceans: More than 5 trillion plastic pieces weighing over 250,000 tons afloat at sea. PLoS ONE.

[21] Ducoli S., Federici S., Cocca M., Gentile G., Zendrini A., Bergese P., Depero L.E. (2024). Characterization of polyethylene terephthalate (PET) and polyamide (PA) true-to-life nanoplastics and their biological interactions. Environ. Pollut..

[22] della Valle M., D’Abrosca G., Gentile M.T., Russo L., Isernia C., Di Gaetano S., Avolio R., Castaldo R., Cocca M., Gentile G. (2022). Polystyrene nanoplastics affect the human ubiquitin structure and ubiquitination in cells: A high-resolution study. Chem. Sci..

[23] Marfella R., Prattichizzo F., Sardu C., Fulgenzi G., Graciotti L., Spadoni T., D’onofrio N., Scisciola L., La Grotta R., Frigé C. (2024). Microplastics and Nanoplastics in Atheromas and Cardiovascular Events. N. Engl. J. Med..

[24] Vethaak A.D., Legler J. (2021). Microplastics and human health. Science.

[25] Smith M., Love D.C., Rochman C.M., Neff R.A. (2018). Microplastics in Seafood and the Implications for Human Health. Curr. Envir. Health Rpt..

[26] Niero M. (2023). Implementation of the European Union’s packaging and packaging waste regulation: A decision support framework combining quantitative environmental sustainability assessment methods and socio-technical approaches. Clean. Waste Syst..

[27] Ambrose C.A., Hooper R., Potter A.K., Singh M.M. (2022). Diversion from landfill: Quality products from valuable plastics. Resour. Conserv. Recycl..

[28] Da S., Paula M.M., Medeiros Rodrigues F.B.B., Bernardin A.M., Fiori M.A., Angioletto E. (2005). Characterization of aluminized polyethylene blends via mechanical recycling. Mater. Sci. Eng. A.

[29] Morris J. (1996). Recycling versus incineration: An energy conservation analysis. J. Hazard. Mater..

[30] Faraca G., Astrup T. (2019). Plastic waste from recycling centres: Characterisation and evaluation of plastic recyclability. Waste Manag..

[31] Capuano R., Bonadies I., Castaldo R., Cocca M., Gentile G., Protopapa A., Avolio R., Errico M.E. (2021). Valorization and Mechanical Recycling of Heterogeneous Post-Consumer Polymer Waste through a Mechano-Chemical Process. Polymers.

[32] Roosen M., Mys N., Kusenberg M., Billen P., Dumoulin A., Dewulf J., Van Geem K.M., Ragaert K., De Meester S. (2020). Detailed Analysis of the Composition of Selected Plastic Packaging Waste Products and Its Implications for Mechanical and Thermochemical Recycling. Environ. Sci. Technol..

[33] Avolio R., Spina F., Gentile G., Cocca M., Avella M., Carfagna C., Tealdo G., Errico M.E. (2019). Recycling Polyethylene-Rich Plastic Waste from Landfill Reclamation: Toward an Enhanced Landfill-Mining Approach. Polymers.

[34] Liotta I., Avolio R., Castaldo R., Gentile G., Ambrogi V., Errico M.E., Cocca M. (2024). Mitigation approach of plastic and microplastic pollution through recycling of fishing nets at the end of life. Process Saf. Environ. Prot..

[35] Bonadies I., Capuano R., Avolio R., Castaldo R., Cocca M., Gentile G., Errico M.E. (2022). Sustainable Cellulose-Aluminum-Plastic Composites from Beverage Cartons Scraps and Recycled Polyethylene. Polymers.

[36] Cappucci G.M., Avolio R., Carfagna C., Cocca M., Gentile G., Scarpellini S., Spina F., Tealdo G., Errico M.E., Ferrari A.M. (2020). Environmental life cycle assessment of the recycling processes of waste plastics recovered by landfill mining. Waste Manag..

[37] Castaldo R., De Falco F., Avolio R., Bossanne E., Cicaroni Fernandes F., Cocca M., Di Pace E., Errico M.E., Gentile G., Jasiński D. (2019). Critical Factors for the Recycling of Different End-of-Life Materials: Wood Wastes, Automotive Shredded Residues, and Dismantled Wind Turbine Blades. Polymers.

[38] Shamsuyeva M., Endres H.-J. (2021). Plastics in the context of the circular economy and sustainable plastics recycling: Comprehensive review on research development, standardization and market. Compos. Part C Open Access.

[39] Eriksen M.K., Astrup T.F. (2019). Characterisation of source-separated, rigid plastic waste and evaluation of recycling initiatives: Effects of product design and source-separation system. Waste Manag..

[40] Jmal H., Bahlouli N., Wagner-Kocher C., Leray D., Ruch F., Munsch J.N., Nardin M. (2018). Influence of the grade on the variability of the mechanical properties of polypropylene waste. Waste Manag..

[41] Lase I.S., Bashirgonbadi A., van Rhijn F., Dewulf J., Ragaert K., Delva L., Roosen M., Brandsma M., Langen M., De Meester S. (2022). Material flow analysis and recycling performance of an improved mechanical recycling process for post-consumer flexible plastics. Waste Manag..

[42] Wilts H., Garcia B.R., Garlito R.G., Gómez L.S., Prieto E.G. (2021). Artificial intelligence in the sorting of municipal waste as an enabler of the circular economy. Resources.

[43] Gupta P.K., Shree V., Hiremath L., Rajendran S. (2019). The use of modern technology in smart waste management and recycling: Artificial intelligence and machine learning. Recent Advances in Computational Intelligence.

[44] Fang B., Yu J., Chen Z., Osman A.I., Farghali M., Ihara I., Hamza E.H., Rooney D.W., Yap P.-S. (2023). Artificial intelligence for waste management in smart cities: A review. Environ. Chem. Lett..

[45] Schmidt J., Marques M.R., Botti S., Marques M.A. (2019). Recent advances and applications of machine learning in solid-state materials science. NPJ Comput. Mater..

[46] Meza J.K.S., Yepes D.O., Rodrigo-Ilarri J., Cassiraga E. (2019). Predictive analysis of urban waste generation for the city of Bogotá, Colombia, through the implementation of decision trees-based machine learning, support vector machines and artificial neural networks. Heliyon.

[47] Roh S.B., Oh S.K., Park E.K., Choi W.Z. (2017). Identification of black plastics realized with the aid of Raman spectroscopy and fuzzy radial basis function neural networks classifier. J. Mater. Cycles Waste Manag..

[48] Erkinay Ozdemir M., Ali Z., Subeshan B., Asmatulu E. (2021). Applying machine learning approach in recycling. J. Mater. Cycles Waste Manag..

[49] Yin S., Tuladhar R., Shi F., Shanks R.A., Combe M., Collister T. (2015). Mechanical reprocessing of polyolefin waste: A review. Polym. Eng. Sci..

[50] Hubo S., Delva L., Van Damme N., Ragaert K. (2016). Blending of recycled mixed polyolefins with recycled polypropylene: Effect on physical and mechanical properties. AIP Conf. Proc..

[51] Miller P., Sbarski I., Kosior E., Masood S., Iovenitti P. (2001). Correlation of rheological and mechanical properties for blends of recycled HDPE and virgin polyolefins. J. Appl. Polym. Sci..

[52] Langwieser J., Schweighuber A., Felgel-Farnholz A., Marschik C., Buchberger W., Fischer J. (2022). Determination of the influence of multiple closed recycling loops on the property profile of different polyolefins. Polymers.

[53] Volfson S.I., Zakirova L.Y., Karaseva Y.S., Nigmatullina A.I. (2019). Effect of the technological additives on the properties of recycled polyolefins. Key Eng. Mater..

[54] Ghosh A. (2021). Performance modifying techniques for recycled thermoplastics. Resour. Conserv. Recycl..

[55] Perugini F., Mastellone M.L., Arena U. (2005). A life cycle assessment of mechanical and feedstock recycling options for management of plastic packaging wastes. Environ. Prog..

